# Supplementary material for: Racial Disparities in the Heavy Metal Contamination of Urban Soil in the Southeastern United States
Source: Int J Environ Res Public Health. 2022 Jan 19;19(3):1105. doi: 10.3390/ijerph19031105 (PMC8834334; doi:10.3390/ijerph19031105)
Supplement: Supplementary file 1 [file ijerph-19-01105-s001.zip › ijerph-1530239-supplementary.pdf]

Supplementary

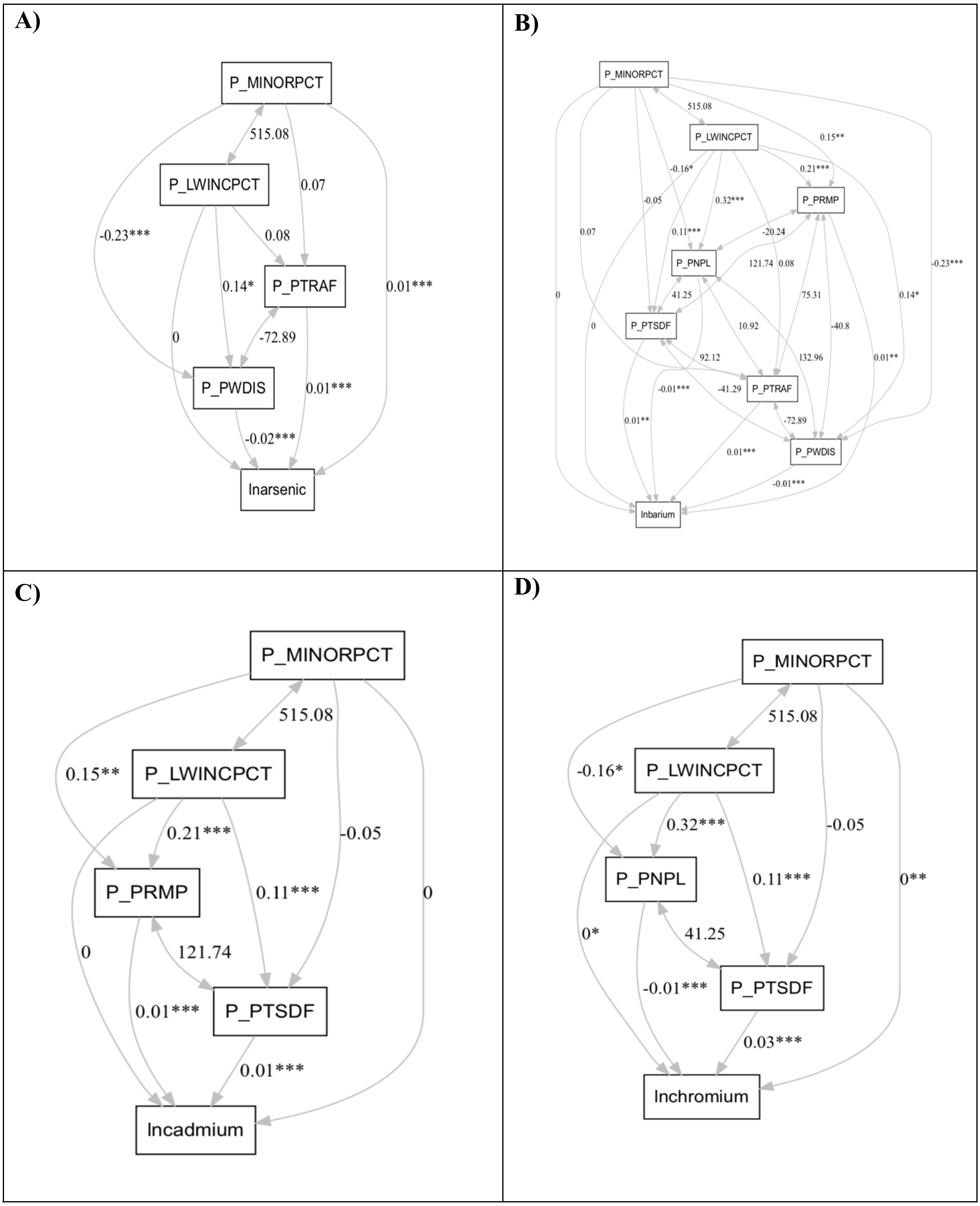

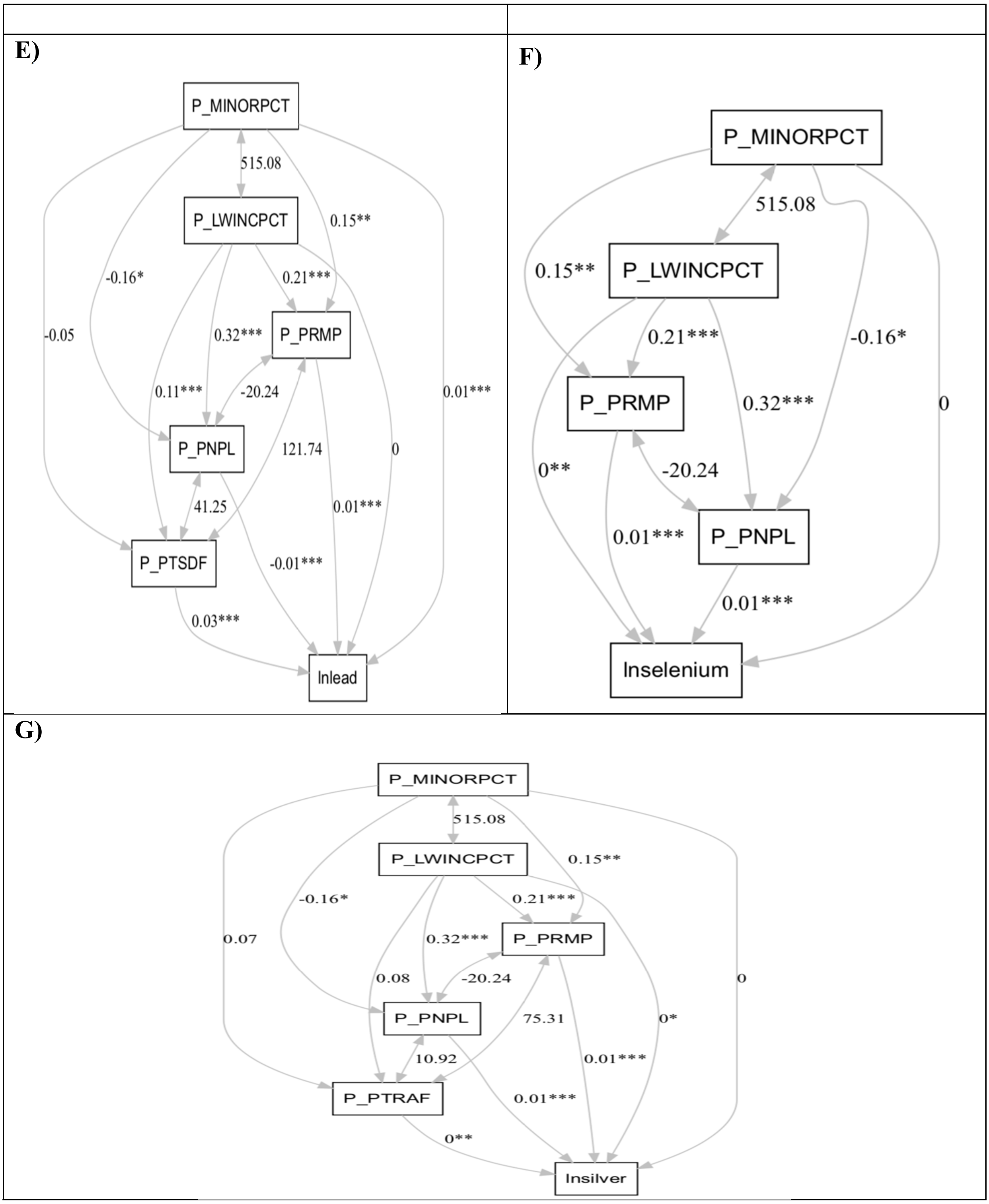

Figure S1. Display of the trimmed path diagrams of heavy metal contamination with the obtained path coefficients, or estimates, of the relationship between the social and environmental factors and soil contamination**. A-G** indicate the heavy metal assessed (arsenic, barium,

cadmium, chromium, lead, selenium, and silver, respectively). The (***) indicate significance at <0.001. The (*) indicate significance at <0.05. **Key:** proximity to traffic (PTRAF), treatment storage and disposal facilities (PTSDF), major and direct discharges to water (PWDIS), national priorities sites (PNPL), and risk management plan facilities (PRMP).

Table S1. Path coefficients of proximity variables in the trimmed models.

	**Traffic Proximity**		**Disposal Proximity**		**Direct Water Discharge**		**National Priorities proximity**		**Risk Management Proximity**	
	β	p	β	p	β	p	β	p	β	p
**As**	**0.18**	**<.0001**	-0.06	0.149	**-0.39**	**<.0001**	0.02	0.629	**0.35**	**<.0001**
**Ba**	**0.23**	**<.0001**	**0.17**	**<.0001**	**-0.20**	**<.0001**	**-0.28**	**<.0001**	**0.14**	**0.002**
**Cd**	0.06	0.171	**0.13**	**0.002**	-0.07	0.102	0.07	0.120	**0.40**	**<.0001**
**Cr**	0.002	0.085	**0.02**	**<.0001**	-0.001	0.593	**-0.01**	**<.0001**	0.001	0.223
**Pb**	0.04	0.297	**0.25**	**<.0001**	**-0.13**	**0.001**	**-0.27**	**<.0001**	**0.25**	**<.0001**
**Se**	**0.11**	**0.008**	0.02	0.728	0.10	0.02	**0.34**	**<.0001**	**0.35**	**<.0001**
**Ag**	**0.14**	**0.003**	0.04	0.423	0.05	0.319	**0.22**	**<.0001**	**0.34**	**<.0001**

Notes: Values are in bold if the p-value is <0.01. Key: Arsenic (As), Barium (Ba), Cadmium (Cd), Chromium (Cr), Lead (Pb), Selenium (Se), and Silver (Ag).

Table S2: Unadjusted association between soil metal contamination and rankings of poverty and minority across cities in Southeastern U.S.

City	Metal	Ranking of Poverty (n=53)			Ranking of Minority (n=53)		
		Estimate[1]	%change[2]	P-value	Estimate[1]	%change[2]	P-value
**Chattanooga**	**As**	1.023	2.3%	0.3853	1.029	2.9%	0.2932
	**Ba**	-1.065	-6.5%	0.0324	-1.070	7.0%	0.0281
	**Cd**	1.025	2.5%	0.1885	1.026	2.6%	0.2062
	**Cr**	-1.004	-0.4%	0.8496	1.009	0.9%	0.7042
	**Pb**	**1.145**	**14.5%**	**0.0025**	**1.185**	**18.5%**	**0.0002**
	**Se**	1.002	0.2%	0.2989	1.002	0.2%	0.2766
	**Ag**	1.001	0.1%	0.5553	1.001	0.1%	0.6439
**Columbia**	**As**	-1.011	-1.1%	0.7775	-1.059	-5.9%	0.0525
	**Ba**	1.094	9.4%	0.0864	-1.064	-6.4%	0.1095
	**Cd**	1.032	3.2%	0.4175	-1.018	-1.8%	0.5475
	**Cr**	1.019	1.9%	0.6481	-1.028	-2.8%	0.3925
	**Pb**	1.080	8.0%	0.2822	-1.074	-7.4%	0.1736
	**Se**	-1.003	-0.3%	0.186	1.001	0.1%	0.5002
	**Ag**	1.010	1.0%	0.7036	1.016	1.6%	0.4345
**Gainesville**	**As**	1.054	5.4%	0.0667	1.067	6.7%	0.1423
	**Ba**	1.006	0.6%	0.8991	-1.130	-13.0%	0.0541
	**Cd**	1.027	2.7%	0.1161	1.019	1.9%	0.4734
	**Cr**	1.034	3.4%	0.2251	-1.042	-4.2%	0.3172
	**Pb**	-1.006	-0.6%	0.8934	1.034	3.4%	0.6198
	**Se**	1.001	0.1%	0.7254	1.003	0.3%	0.4114
	**Ag**	1.017	1.7%	0.2232	-1.012	-1.2%	0.5888
**Lexington**	**As**	1.021	2.1%	0.0867	1.016	1.6%	0.2729
	**Ba**	1.003	0.3%	0.8661	-1.031	-3.1%	0.1868
	**Cd**	1.058	5.8%	0.015	1.010	1.0%	0.7123
	**Cr**	1.004	0.4%	0.6797	1.015	1.5%	0.2032
	**Pb**	**1.130**	**13.0%**	**0.001**	1.041	4.1%	0.3691
	**Se**	1.001	0.1	0.8464	1.001	0.1%	0.9156
	**Ag**	**1.044**	**4.4%**	**<.0001**	1.025	2.5%	0.0607
**Louisville**	**As**	1.008	0.8%	0.6476	-1.008	-0.8%	0.6839
	**Ba**	-1.007	-0.7%	0.7787	-1.037	-3.7%	0.1858
	**Cd**	1.047	4.7%	0.2078	1.062	6.2%	0.1333
	**Cr**	-1.012	-1.2%	0.5152	1.016	1.6%	0.4096
	**Pb**	1.003	0.3%	0.9614	1.035	3.5%	0.554

City	Metal	Ranking of Poverty (n=53)			Ranking of Minority (n=53)		
		Estimate[1]	%change[2]	P-value	Estimate[1]	%change[2]	P-value
	**Se**	-1.034	-3.4%	0.0066	-1.026	-2.6%	0.0652
	**Ag**	-1.033	-3.3%	0.4325	-1.047	-4.7%	0.3079
**Memphis**	**As**	**-1.171**	**-17.1**	**0.0037**	-1.071	-7.1%	0.2427
	**Ba**	1.013	1.3%	0.7117	1.019	1.9%	0.5602
	**Cd**	1.050	5.0%	0.4151	1.061	6.1%	0.2881
	**Cr**	1.023	2.3%	0.3065	-1.015	-1.5%	0.4664
	**Pb**	-1.080	-8.0%	0.2882	1.160	16.0%	0.0411
	**Se**	-1.022	-2.2%	0.0736	-1.012	-1.2%	0.2974
	**Ag**	1.021	2.1%	0.5199	1.038	3.8%	0.2133
**Raleigh**	**As**	-1.002	-0.2%	0.9558	1.005	0.5%	0.8779
	**Ba**	1.009	0.9%	0.7413	-1.010	-1.0%	0.6912
	**Cd**	1.031	3.1%	0.0866	1.026	2.6%	0.1316
	**Cr**	1.017	1.7%	0.4466	1.002	0.2%	0.927
	**Pb**	1.051	5.1%	0.1663	1.021	2.1%	0.5544
	**Se**	-1.005	-0.5%	0.7802	-1.029	-2.9%	0.0672
	**Ag**	1.031	3.1%	0.2652	1.029	2.9%	0.2799
**Winston Salem**	**As**	-1.075	-7.5%	0.0434	-1.066	-6.6%	0.0771
	**Ba**	1.009	0.9%	0.6569	-1.008	-0.8%	0.6725
	**Cd**	-1.027	-2.7%	0.2705	-1.017	-1.7%	0.4872
	**Cr**	-1.017	-1.7%	0.3383	-1.011	-1.1%	0.5205
	**Pb**	-1.018	-1.8%	0.6837	1.007	0.7%	0.8729
	**Se**	1.000	0%	0.8826	1.000	0%	0.4251
	**Ag**	-1.040	-4.0%	0.1337	-1.041	-4.1%	0.1208

Notes: 1. Estimate is $\beta1$ from the crude model: log [Metal Concentration] = $\beta0 + \beta1 \times$Ranking of Poverty (or Minority). $\beta1 > 0$ suggests a positive association, and vice versa. 2. Percent change means percent change in the metal concentration per 10-percentile change in the ranking of poverty or minority. 3. Values are in bold if the p-value is <0.01. Key: Arsenic (As), Barium (Ba), Cadmium (Cd), Chromium (Cr), Lead (Pb), Selenium (Se), and Silver (Ag).
